# An electrically conductive dinuclear aluminium complex for the fabrication of a Schottky diode

**DOI:** 10.1039/d4ra07123a

**Published:** 2025-01-22

**Authors:** Md. Akhtarul Alam, Anamika Hoque, Md Sanaul Islam, Nargis Khatun, Manash Pratim Sarmah, A. K. M. Maidul Islam, Manabendra Sarma, Goutam Kumar Kole, Ennio Zangrando

**Affiliations:** a Department of Chemistry, Aliah University Action Area IIA/27, New Town Kolkata 700160 India alam_iitg@yahoo.com; b Department of Physics, Aliah University Action Area IIA/27, New Town Kolkata 700160 India maidul79@gmail.com; c Department of Chemistry, Indian Institute of Technology Guwahati Assam 781039 India msarma@iitg.ac.in; d Department of Chemistry, SRM Institute of Science and Technology Kattankulathur Tamil Nadu 603203 India goutamks@srmist.edu.i; e Department of Chemical and Pharmaceutical Sciences, University of Trieste Via L. Giorgieri 1 Trieste 34127 Italy ezangrando@units.it

## Abstract

Electrical performances of a biphenyl-derived amido Schiff base ligand L and its dinuclear Al(iii) complex (complex 1) were investigated in a metal–semiconductor (MS) junction. Electrical studies revealed that complex 1 significantly enhanced the electrical conductivity and improved the characteristics of a Schottky barrier diode (SBD). The *I*–*V* characteristics demonstrated that complexation of ligand L with Al(iii) ion increased the conductivity by two orders of magnitude (conductivity of L = 1.04 × 10^−7^ Sm^−1^ and complex 1 = 1.04 × 10^−5^ Sm^−1^) with improved diode rectification ratio. Complex 1 extended itself to the 3D supramolecular array by virtue of the hydrogen bond, C–H⋯π(C) bond and π⋯π interactions. This significantly influenced the semiconducting behaviour of complex 1 and essentially improved the characteristics of SBD. The optical band gap of complex 1 and ligand L in the solid state was determined experimentally (2.63 eV and 3.04 eV, respectively) and compared with the theoretical value obtained from DFT calculations. Furthermore, DOS analysis explained the conductivity behavior of complex 1 in a logically better way.

## Abstract 2

The dimeric Al(iii) complex 1 generated 1D polymeric chain *via* the C–H⋯π bond and π⋯π interactions and significantly influenced in its Schottky barrier diode behaviour.

## Graphical abstract

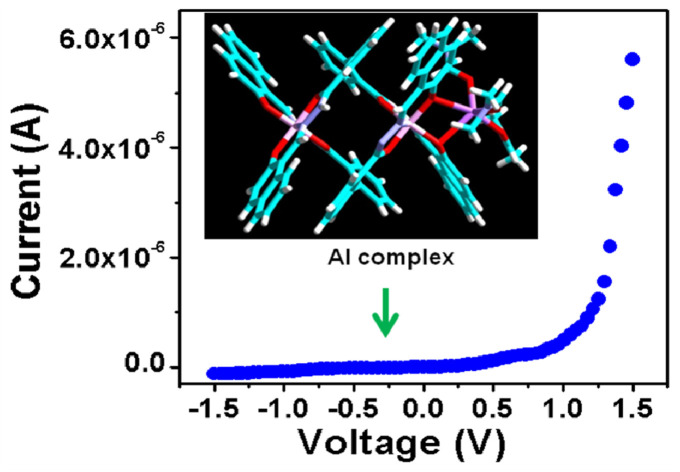

## 1. Introduction

In the last few decades, chemists have paid a lot of attention towards synthetic metal–organic hybrid compounds for the fabrication of electronic and optoelectronic devices.^1–3^ The electrical properties of such synthetic metal–organic hybrid materials^4,5^ depend mainly on (a) the nature of the bonding between the metals and organic moieties containing chelating functional groups within the structural architecture of the metal–organic hybrid complex and (b) the extent of conjugation between the organic and metal components.^6,7^ Thus, the chemical and physical properties of such metal–organic hybrid materials can be easily fine-tuned simply by varying its chemical structure (both the metal centre and/or organic ligand)^8,9^ to develop a suitable material to fit a particular application.^10^

In this regard, metal–organic hybrid compounds^11,12^ made of Schiff base ligands have attained a significant interest owing to their attractive chemical and physical properties and their wide range of applications in various fields.^13,14^

The fundamental routes for the syntheses of Schiff base as ligands are straightforward and cost-efficient.^15^ Similarly, the preparation of Schiff base-containing metal complexes are also simple and less expensive.^16^ In addition to this, slight modification by incorporating different substituent groups within the structural motif of Schiff base ligands results in the modulation of various significant characteristics of the metal complexes. Owing to these reasons, Schiff base-containing metal complexes are considered an ideal choice for the fabrication of electronic devices.^17–19^ Besides, the large-scale production of the Schiff base-containing metal complexes is reasonably easy and their industrial implementation in the fabrication of electronic devices is highly appreciable.

Recently, several research groups have developed new strategies for the fabrication of electronic devices using Schiff base metal complexes. For example, S. Chattopadhyay *et al.* reported that cadmium(ii)- and copper(ii)-containing Schiff base complexes can be used as a conductivity-based photo-switching device.^20–22^ Y.-B. Dong *et al.* reported that Ag(i) complexes containing double Schiff base ligands can exhibit luminescent and electrical conductive properties.^23^ S. Banerjee *et al.* reported a binuclear copper(ii) Schiff base complex that exhibits electrical properties.^18^ D. Majumdar *et al.* reported that Schiff base ligand and its Cd(ii)-based coordination polymer can be used to construct a photosensitive Schottky barrier diode.^24^ Saha *et al.* reported another novel Cd(ii)-Schiff base complex that exhibits photosensitive Schottky diode behaviour.^25^ Besides, several hetero-metal complexes^26^ containing Schiff base as ligand have also been used to fabricate devices with interesting electrical conducting properties.^27,28^

Although many metal complexes consisting of Schiff base as ligands have been used to fabricate photosensitive Schottky barrier diodes, electrical conductivity, semiconductor devices, *etc.*, to the best of our knowledge, there is no report in the literature to use any amido Schiff base containing aluminium(iii) complex for the fabrication of electronic device till date.

We have been exploring the metal–organic assemblies based on the amide group containing the Schiff base ligand.^29,30^ Our previous research demonstrated that an amide-based Schiff base ligand, bis(2-hydroxynaphthalen-1-yl)methylene)-[1,1′-biphenyl]-2,2′-dicarbohydrazide (ligand L) can selectivity and sensitively detect Al^3+^ ions in aqueous DMF media. The crystal structure of ligand L and its aluminium complex (complex 1) have also been reported.^31^

Pursuing our research in this area, we investigate the electrical conducting properties of ligand L and its Al(iii) complex (complex 1). Importantly, the Al(iii) complex (complex 1) containing an amide-based Schiff base ligand L exhibits Schottky barrier diode behaviour. As complex 1 is connected with its neighbour complexes through hydrogen bonds, C–H⋯π(C) bond and π⋯π interact to generate a polymeric chain, which might have significant influences on the electrical conductivity. The optical band gap of complex 1 and ligand L have also been determined by experimental measurements and compared with the theoretical values obtained from DFT calculations, which indicates that complex 1 exhibits Schottky barrier diode behaviour.

## 2. Experimental section

### 2.1. Theoretical methods

All the optimization calculations were performed with the DFT-B3LYP/6-311G(d) level at the Gaussian-16 software.^32^

DOS/PDOS calculations were performed using the Quantum ESPRESSO^33^ software to analyze the conductivity of the compounds. The Perdew–Burke–Ernzerhof (PBE) function with generalized gradient approximation (GGA) approximates the exchange-correlation terms.^34^ Projector augmented wave (PAW) pseudo potential was used to treat the electron-ion core interaction, and the cut-off energy for the plane wave was maintained at 200 eV. For the self-consistent field (SCF) calculation, the Brillouin zone was sampled at 3 × 3 × 3; for the DOS calculation, it was sampled at 6 × 6 × 6.

### 2.2. Device fabrication

**Scheme 1:**
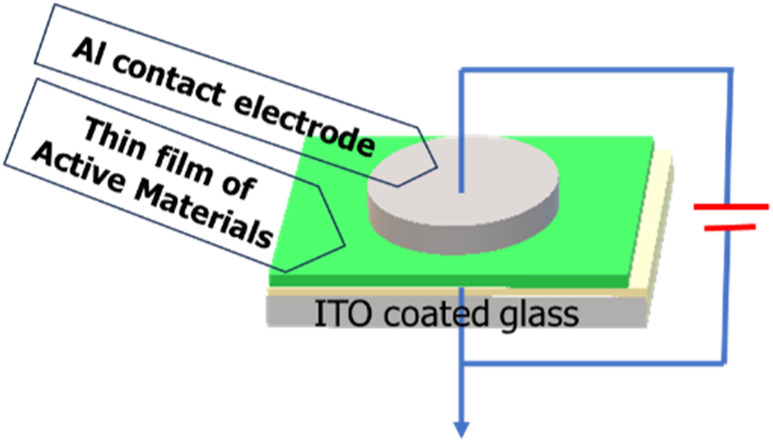
Schematic of metal–semiconductor junction for electrical measurements.

For the electrical measurements, a metal–semiconductor (MS) junction device was fabricated using an indium tin oxide (ITO) electrode in an ITO/active material/Al sandwich structure. The active material pertains to our synthesized ligand L and its Al(iii) complex (complex 1). Two devices were prepared for the comparative study, one incorporating complex 1 and the other using ligand L. For this purpose, a homogeneous dispersion of ligand L or complex 1 was prepared in *N*,*N*-dimethyl formamide (DMF) solvent as the dispersive medium by dissolving 5 mg of material in 1 mL of the solvent. Afterwards, these freshly prepared stable, well-dispersed materials were drop cast onto a precleaned ITO-coated glass substrate to prepare an approximately ∼10 μm thick thin film. ITO-coated glass substrates were obtained and sequentially cleaned by sonication with isopropanol, acetone and deionized water in an ultrasonic bath for 10 min each. The film was then dried in a vacuum oven at 5 × 10^−3^ m bar and 75 °C for the evaporation of the solvent. The aluminium (Al) electrode was deposited on top of the films using the thermal evaporation (advanced process technology) technique. The effective area of the electrode as 1.95 × 10^−5^ m^2^ was determined by the deposition through a shadow mask. The base pressure of 5 × 10^−6^ mbar was maintained during the metal deposition. The metal–semiconductor (MS) junction Schottky diode preparation was finally completed in an ITO/ligand L or complex 1/Al sandwich pattern, as shown in Scheme 1, where Al was employed as the metal electrode.

Electrical characterization of the devices was performed by measuring the current–voltage (*I*–*V*) characteristics with the help of a Keysight B2902A source meter using the two-probe technique. All preparation and measurements were performed at room temperature and under ambient conditions.

## 3. Results and discussion

**Fig. 1:**
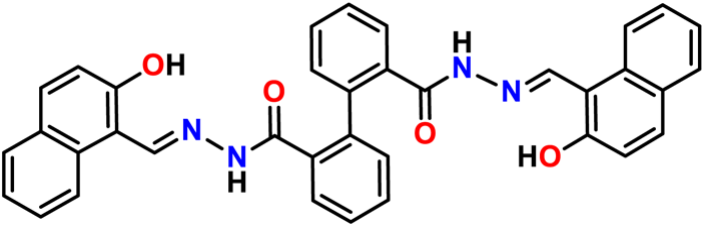
Molecular structure of ligand L.

Syntheses^29^ and single crystal structure of amido Schiff base containing ligand L (Fig. 1) and its aluminium complex containing heterometallic atoms (complex 1, CCDC: 2260348) with two distinct Al^3+^ ion and one Na^+^ ion [([Na(Al_2_L_2_)·2H_2_O·3.5DMF] have been reported by our group.^31^

**Fig. 2:**
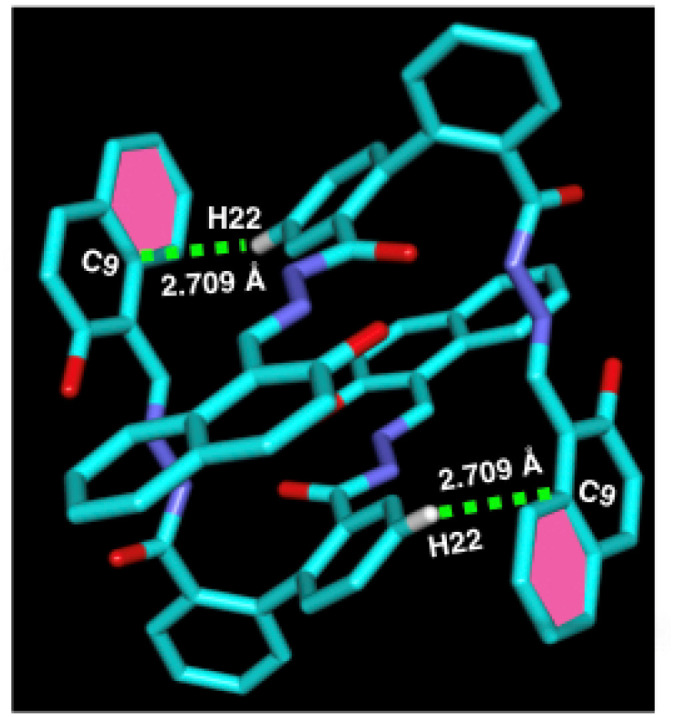
Perspective views of C–H⋯π interactions of complex 1.

In the crystal structure of ligand L (CCDC: 2260347, Fig. 2), one molecule containing phenyl ring H22 attached to C22 is involved in a strong intermolecular C–H⋯π(C) interaction^35,36^ with the naphthyl ring containing (C4–C9) of the adjacent molecule (distance 3.551 Å, Table 1) and *vice versa* to form a dimer (Fig. 2). Besides a weak π⋯π interaction within the adjacent naphthyl ring Cg(C26–C27) of a distance at 3.390 Å was observed (Table 1).

Geometric parameters (distances in Å and angles (°)) of intermolecular interactions for the complex 1 and the ligand L

**Table:** 

C–H⋯π(C)	C⋯π(C) distance (Å)	H⋯π(C) distance (Å)	C–H⋯π(C) angle (°)
**Complex 1**			
C17–H17⋯Cg(C30–C31)	3.576	2.769	145.65
C21–H21⋯C67	3.734	2.831	164.24
C31–H31⋯C16	3.622	2.888	136.82
C67–H67⋯C39	3.771	2.975	144.54

**Ligand L**			
C22–H22⋯C9	3.551	2.709	148.08

**Table:** 

π⋯π interaction	Distance (Å)
**Complex 1**	
Cg(C23–C24–C25)⋯Cg(C29–C28–C33)	3.396–3.461

**Ligand L**	
Cg(C26–C27)⋯Cg(C27–C26)	3.390

**Fig. 3:**
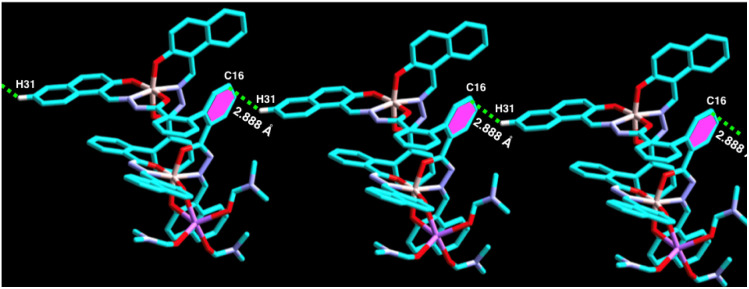
Perspective views of C–H⋯π(C) interactions of complex 1.

On the other hand, in complex 1, the naphthyl ring containing hydrogen atom, H31 attached to C31 is involved in strong intermolecular C–H⋯π(C) interaction with π ring of the biphenyl containing one of the phenyl moiety of the adjacent molecule (distance 3.622 Å, Fig. 3 and Table 1). Thus, complex 1 forms 1D supramolecular chain *via* this C–H⋯π(C) interaction. Furthermore, C21–H21⋯π(C) interaction (*d*_C–C_ ∼ 3.576 Å), C67–H67⋯π(C) interaction (*d*_C–C_ ∼ 3.771 Å), and C17–H17⋯Cg(C30–C31) interaction (*d*_C–C_ ∼ 3.576 Å) have also been observed (Table 1) and form a 2D supramolecular structure.

**Fig. 4:**
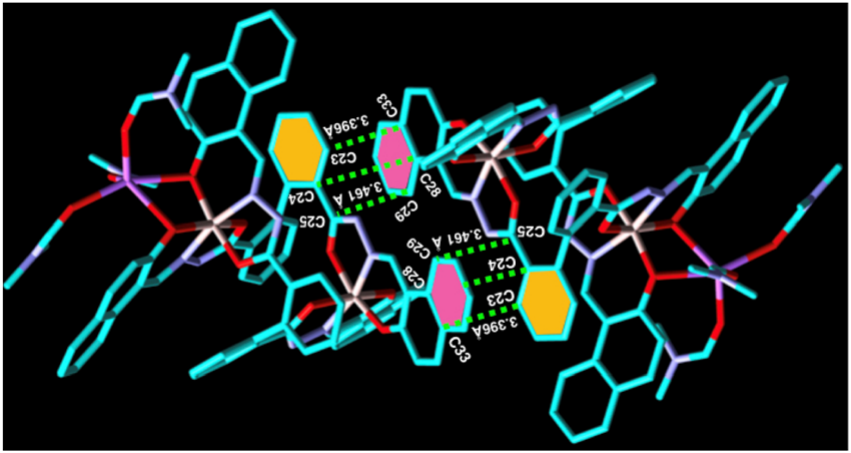
Perspective views of π⋯π interactions between two adjacent complex 1.

Moreover, the biphenyl-containing phenyl ring Cg(C23–C24–C25) is involved in strong π⋯π interaction with the adjacent naphthyl ring Cg(C28–C29–C33C) within a distance of 3.396 Å–3.461 Å and *vice versa* (Fig. 4 and Table 1), which further stabilised the supramolecular chain. Thus, ligand L can form a dimeric structure through strong C–H⋯π interaction. On the other hand, complex 1 can form a 3D polymeric structure, which is stabilised by strong C–H⋯π interaction and π⋯π interactions. Thus, it is expected that complex 1 might show some electrical conductivity.

## 4. Electrical characterisation: *I*–*V* analysis

**Fig. 5:**
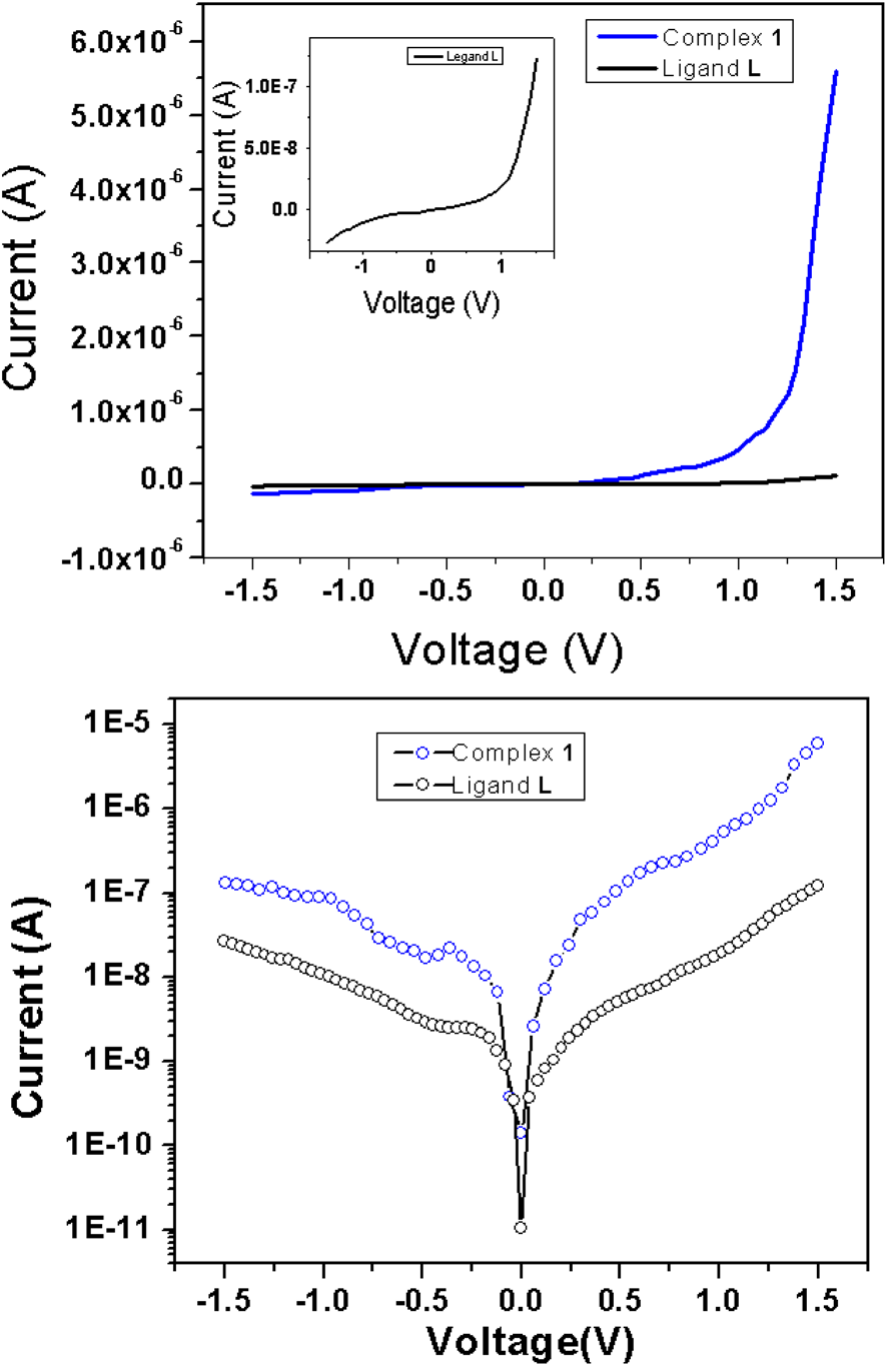
*I*–*V* characteristics of Al/complex 1/ITO and Al/ligand L/ITO (for better clarity it is also shown in inset) in (top) linear scale and (bottom) in semi-log scale.

As evident from the UV-vis studies and DFT calculations, the suitable band gap suggests that ligand L, with Al complexation, could be a promising candidate for semiconductor materials. The supramolecular chain structure of complex 1, stabilized through C–H⋯π interactions, further underscores its potential for electronic devices. To investigate their performances, we have performed electrical characterization of thin film devices with a metal (Al) and semiconductor (MS) junction. For this purpose, Schottky diodes were fabricated in an Al/active material/ITO configuration using previously described ligand L or complex 1 as the active material. To evaluate the various junction features, including the ideality factor (*η*), rectification ratio (RR), reverse saturation current, barrier height (*φ*_B_) and series resistance (*R*_S_), we conducted the current–voltage (*I*–*V*) measurements of both the Schottky diodes. The data were collected under biased conditions, ranging from −1.5 to +1.5 volts, as shown in Fig. 5. The top panel of Fig. 5 displays the data on a linear scale, while the bottom panel presents it on a semi-logarithmic scale. Both materials exhibit an exponential growth in current when a forward bias is applied. However, the voltage dependency decreases in a reversed biased condition. Notably, the Al(iii) complex (complex 1) generates approximately two orders of magnitude more current than its ligand L counterpart, indicating a significant increase in conductivity resulting from metal insertion in the ligand L. Incorporating the metal into the ligand facilitates the formation of a one-dimensional supramolecular architecture *via* C–H⋯π interactions, enhancing overall conductivity and promoting more efficient electron transfer relative to ligand L. The improved conductivity of complex 1 is also ascribed to its optimal band gap, as demonstrated by UV-vis analyses and DFT computations. The conductivity estimations derived from the forward bias *I*–*V* characteristic give early insights into the junction's behaviour. They yield conductivity values of 1.04 × 10^−5^ Sm^−1^ and 1.05 × 10^−7^ Sm^−1^ for the complex 1 and ligand L, respectively (Table 2). The findings support the typical semiconductor properties of the materials and reveal a possible increase in conductivity for the metal complex. When presented on a semi-logarithmic scale, the current–voltage (*I*–*V*) characteristics reveal an asymmetrical pattern, exponential growth occurs with a forward bias, and the reduced current is observed when a reverse bias is applied. These features evoke the rectifying properties generally observed in Schottky barrier diodes (SBD). To quantify this behaviour, we evaluated the rectification ratio by dividing the forward bias current by the reverse bias current (*I*_on_/*I*_off_) for a particular applied voltage. The rectification ratio (*I*_on_/*I*_off_) for the Schottky barrier diodes (SBD) at 1.5 volts was found to be 5.27 and 42 for ligand L and complex 1, respectively, which shows better rectification for the metal complex-based diode. This is consistent with earlier investigations of similar materials that are likely related to the formation of an enhanced space charge layer at the contact for the complex 1 in contrast to the ligand L.

**Table 2:** Schottky diode parameters of complex 1 and ligand L

Compounds	Conductivity (S m^−1^)	Ideality factor	Rectification ratio	*R*_s_ from d*V*/d ln *I* (MΩ)	*R*_s_ from *H*(*I*) (MΩ)	Barrier height from *H*(*I*)
Complex 1	1.04 × 10^−5^	1.54	42	3.84	2.89	0.85
Ligand L	1.05 × 10^−7^	2.31	5.27	55.7	57.4	0.95

A more detailed analysis of *I*–*V* characteristics of Schottky barrier diodes (SBD) was conducted using the thermionic emission (TE) theory for more profound insight. This theory is the most suitable model for explaining current emissions at the metal–semiconductor (M–S) interface. In this context, the *I*–*V* curves were quantitatively analyzed using the following standard equations.^37^1
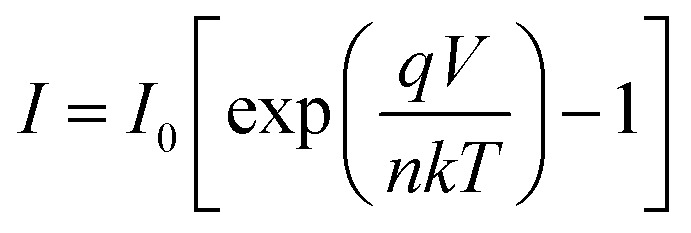
where,2
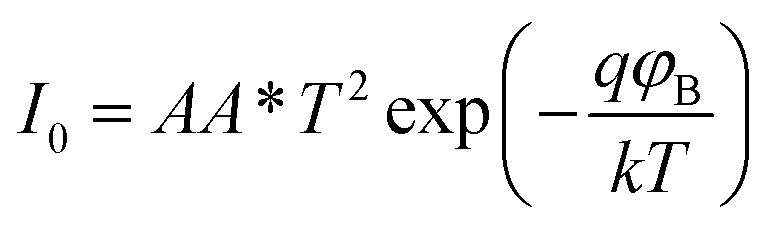
and3
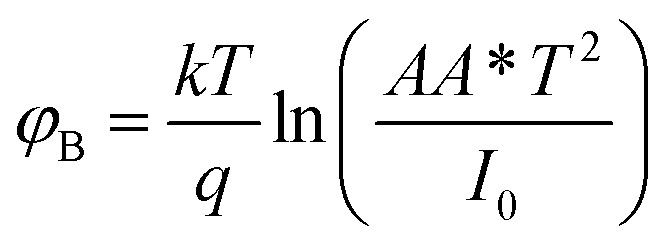
*I*_0_, *q*, *k*, *T*, *A*, A*, *η*, and *φ*_B_ represent the reverse saturation current, electronic charge, Boltzmann constant, temperature in Kelvin, effective diode area, effective Richardson constant, ideality factor, and the effective barrier height at zero bias, respectively.

**Fig. 6:**
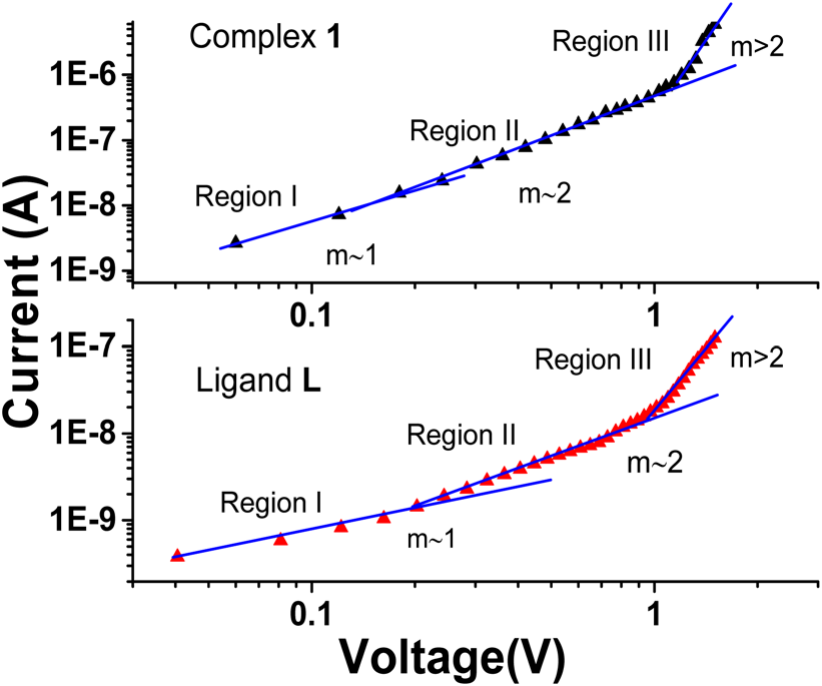
Logarithmic plot of *I*–*V* characteristics of Al/complex 1/ITO and Al/ligand L/ITO structured device.

To examine different conduction mechanisms, the *I*–*V* data was re-plotted on a double logarithmic scale (ln *I vs.* ln *V*) and analysed using a linear regression technique, as illustrated in Fig. 6. Commonly demonstrating power law behaviour I ∞ *V*^*m*^, the double logarithmic forward bias *I*–*V* plot characterizes different conduction mechanisms, where the slope “*m*” dictates the different conduction regions. A value of “*m*” equal to 1 correlates to ohmic behaviour, whereas a value of 2 implies space-charge-limited current (SCLC). Values of “*m*” greater than 2 indicate the trapped-charge limited-current region. In different conduction mechanisms, “*m*” depends on the injection level and is associated with the distribution of trapping centres, ultimately determining specific conduction pathways.^38–40^ The ln *I vs.* ln *V* graph, as shown in Fig. 6, clearly exhibits non-linearity following the power law, *I* ∝ *V*^*m*^, for both the complex 1 and ligand L-based devices. This graph reveals three distinct regions under forward bias conditions.

In the first region, the slope *m* is found to be 1.45 and 0.87 for complex 1 and ligand L-based devices, respectively, which can approximately indicate ohmic conduction (*I* ∝ *V*). In this region, a gradual increase in current with applied voltage is observed, and the *I*–*V* characteristics can be attributed to thermionic emission. This behaviour is due to the fact that the current in this region is primarily influenced by bulk-generated electrons within the active thin film, as the injected effective charge carrier density is lower than the background thermal carrier density.^41–43^

The second region has a slope of 2.04 for the device based on complex 1, which is considerably higher than that of the ligand L-based device with a slope of 1.87. However, both samples indicate a quadratic current dependency on voltage, with small voltage changes having a more meaningful impact than in the first region. This spike in current is ascribed to the density of injected free charges exceeding the density of thermally generated free-charge carriers, leading to the creation of Space Charge Limited Current (SCLC). The existence of a space charge field generates the increased current. In particular, the enhanced performance of the device based on complex 1 demonstrates the higher quantity of injected carriers compared to its ligand counterpart.^44^

Subsequently, in region 3, a sizeable exponential increase in current is observed at higher voltages, resulting in a slope greater than 2.0 (approximately 6.02) for the complex 1-based device, suggesting exponential charge development(*I* ∞ *V*^*m*^). The trap-filled limit fundamentally governs this behaviour (TFL), wherein all deep traps are occupied by injected electrons, resulting in full occupancy of the accessible trap sites. Consequently, with increasing voltage, more charges get exponentially trapped, hence the term ‘exponential trapped region. This result illustrates the complex charge transport dynamics at higher voltage levels in the complex 1-based device.

To determine different Schottky parameters, such as the ideality factor (*η*), barrier height, and series resistance (*R*_S_), we explored the linear ohmic region of the current–voltage (*I*–*V*) characteristics. Fig. 5 (top) shows that the current's linearity at lower voltages deviates at higher voltages is likely due to the presence of series resistance (*R*_S_) in the metal–semiconductor junction, which is visible as downward curvature in the forward bias ln(*I*)–*V* characteristics (Fig. 5 (bottom)). By utilising Cheung's equations, we addressed the influence of *R*_S_ and determined two important Schottky parameters, including the barrier height (*Φ*_B_) and ideality factor (*η*).^45,46^ The implementation of Cheung's technique entails the utilization of relevant mathematical functions as follows.4
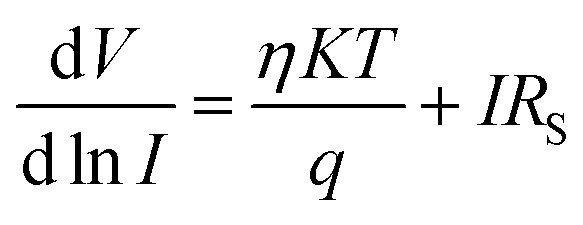
5
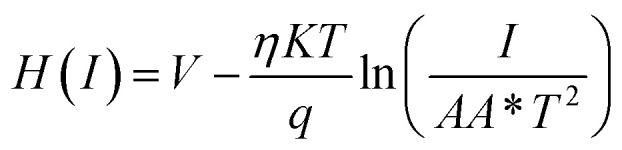
where *H*(*I*) can be written as:

6 *H*(*I*) = *ηΦ*_B_ + *IR*_S_

**Fig. 7:**
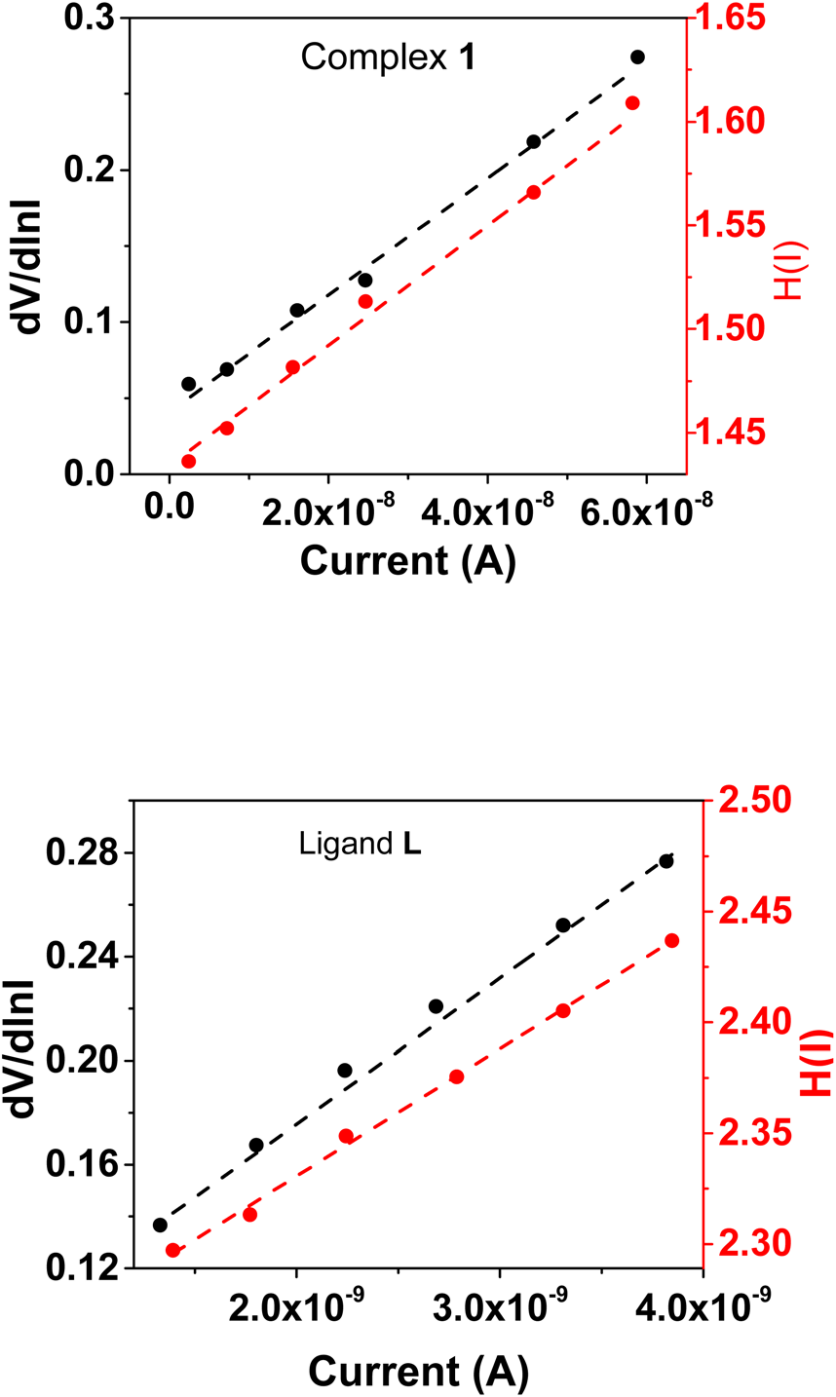
(a) d*V*/d ln *I vs. I* and *H*(*I*)*vs. I* graphs for complex 1. (b) d*V*/d ln *I vs. I* and *H*(*I*)*vs. I* graphs for ligand L.

Eqn (4) is expected to yield a linear relationship when applied to the data from the region of downward curvature in the forward bias ln(*I*)–*V* characteristics, as shown in Fig. 5 (bottom). Consequently, from the d*V*/d *ln*(*I*)*vs. I* plot, as shown in Fig. 7a, the two key parameters, *R*_S_ (series resistance) and the ideality factor *η*, can be calculated. The *Y*-axis intercept and the slope of the d*V*/d ln(*I*)*vs. I* graph (Fig. 7b) will provide the values of the ideality factor and series resistance of the device correspondingly mentioned in Table 2. The values are determined to be 1.54 and 2.31 for complex 1 and the ligand L-based device, respectively. The results suggest an improvement of the diode property of complex 1 over the ligand L-based diode, which demonstrated a lower ideality factor. However, the ideality factor deviates from unity for both diodes, which should be the case for an ideal diode. This difference is likely attributed to the existence of interface states, structural defects, inhomogeneities at the M–S junction, variations in the Schottky barrier height, and the influence of high series resistance(*R*_S_).^47^

It is worth noting that the complex 1-based device has improved and become more ideal than the ligand L-based diode. This may be due to a decrease in the recombination of charges at the interface and an improvement in the homogeneity of Schottky junctions after converting the ligand L into its organometallic complex.

Using the *η* value from eqn (4) and the data from the downward-curvature section of the semi-logarithmic forward bias *I*–*V* characteristics in eqn (5), we can generate a plot of *H*(*I*)*vs. I* using eqn (6). This plot, with a *Y*-axis intercept equal to *ηφ*_B_, illustrates a linear relationship. The slope of this plot presents an alternate technique to compute the series resistance (*R*_S_), which helps verify the consistency of Cheung's method. The series resistance values derived from both methods are presented in Table 2, and the associated graphs are displayed in Fig. 7. The consistency of the series resistance values from the two Cheung plots, together with the *H*(*I*)–*I* plots and the d*V*/d ln(*I*)–*I* diagrams, validates the reliability of our results.^45,48^

Fascinatingly, compared to the diode made of ligand L, the diode constructed with complex 1 showed a lower series resistance and barrier height. This improvement can likely be attributed to the incorporation of metal, which facilitates the formation of supramolecular structure of the molecules where hydrogen bond, C–H⋯π(C) bond and π⋯π interactions enhances overall conductivity, enabling more efficient electron transfer relative to ligand L. As observed earlier, the superior conductivity of complex 1 is further supported by its optimal band gap, as confirmed by UV-vis analyses and DFT calculations and hence reduces both series resistance and barrier height relative to its ligand L counterpart. Moreover, the surface conditions and interface quality between the metals (Al^3+)^ and semiconductors greatly affected the device properties. The improved performance of devices with complex 1 underscores our material's possible applicability in organic electronics. Our study showed that both the metal–semiconductor interface quality and surface conditions are important in determining the device characteristics.^43,45^

## 5. UV-vis spectroscopy and band gap

**Fig. 8:**
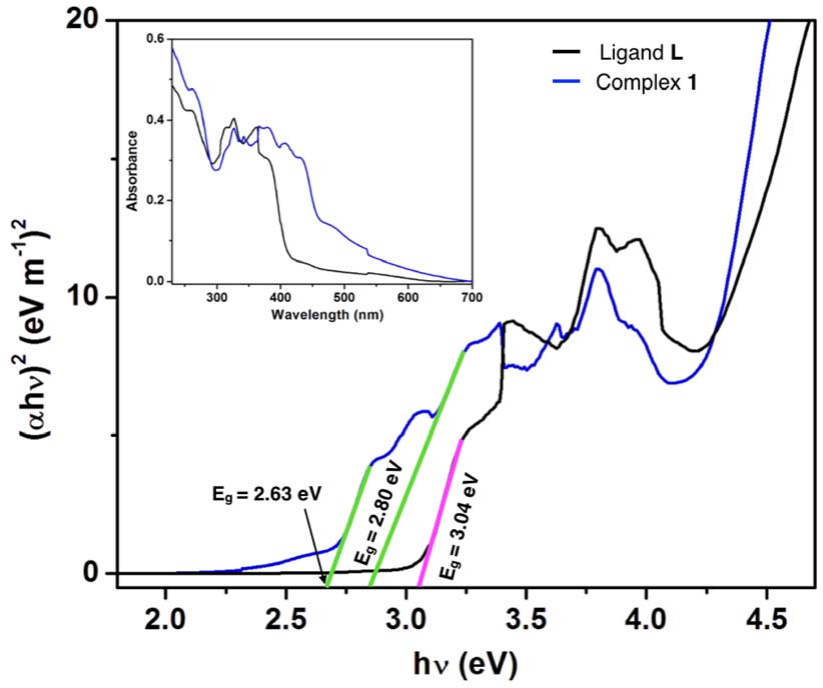
UV-vis absorption spectra (inset) and Tauc plots for ligand L and complex 1.

We measured the UV-vis absorbance spectra of ligand L and its Al(iii) complex (complex 1) to ascertain the optical band gap.^24^ The inset of Fig. 8 displays the UV-vis absorption spectra of the ligand L and complex 1 in the range of 230–700 nm. The optical band gaps (*E*_g_) of the ligand L and complex 1 were determined with the help of Tauc's equation, which corresponds to the electronic transition from the valence band to the conduction band.^49–51^ The Tauc's equation, we have used,

7 (*αhν*)^*n*^ = *A*(*hν* −*E*_g_)

where *α*, *E*_g_, *h*, and *ν* are the absorption coefficient, optical band gap energy, Planck's constant and frequency, respectively. Here, *A* is an arbitrary constant which is 1 for the ideal case, and *n* is the order of energy transition. The values of *n* were taken as 2 and ½, corresponding to the allowed direct and indirect electronic transitions, respectively. By extrapolating the linear region of the plot (*αhν*)^2^*vs. hν* to *α* = 0 absorption, the values of direct optical band gap (*E*_g_) of the complex 1 were evaluated as 2.63 eV and 2.80 eV and for the ligand L it was 3.04 eV (Fig. 8 and Table 3). Such optical band gap of complex 1 clearly suggests the semi-conducting property.^52^

**Table 3:** Values of optical energy gaps of ligand (L) and its Al(iii) complex (complex 1)

Compounds	Energy gap (eV)
Ligand L	3.04
Complex 1	2.63 and 2.80

## 6. DFT calculation and HOMO–LUMO energy gap analysis

**Fig. 9:**
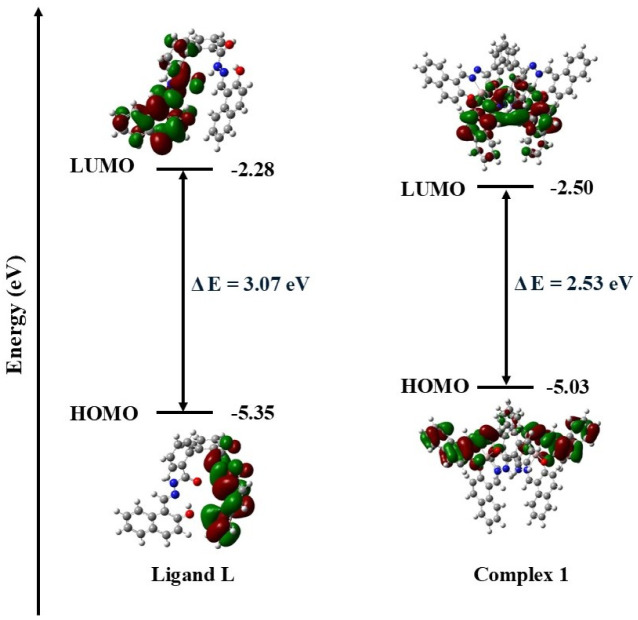
HOMO, LUMO, and HOMO–LUMO energy gaps for ligand L and complex 1 using B3LYP/6-311G(d) level of theory.

To obtain the theoretical insight into the complex 1, DFT calculations were performed with the DFT-B3LYP/6-311G(d) level at the Gaussian-16 software.^32^ During the evaluation of the HOMO–LUMO band gap, single point calculation was performed for complex 1 from where the corresponding energies and orbital diagrams with electron density were calculated. The energy values of HOMO and LUMO for ligand L and its Al(iii) complex, complex 1, are shown in Fig. 9. Complex 1 has a lower HOMO–LUMO gap compared to the ligand L. We observed the HOMO–LUMO gap of ligand L and complex 1 as 3.07 eV and 2.53 eV respectively. These values are almost close to the band gaps obtained from Tauc plot (Table 3). The HOMO–LUMO gap also indicates that complex 1 exhibits greater conductivity than ligand L due to its smaller HOMO–LUMO gap.

**Fig. 10:**
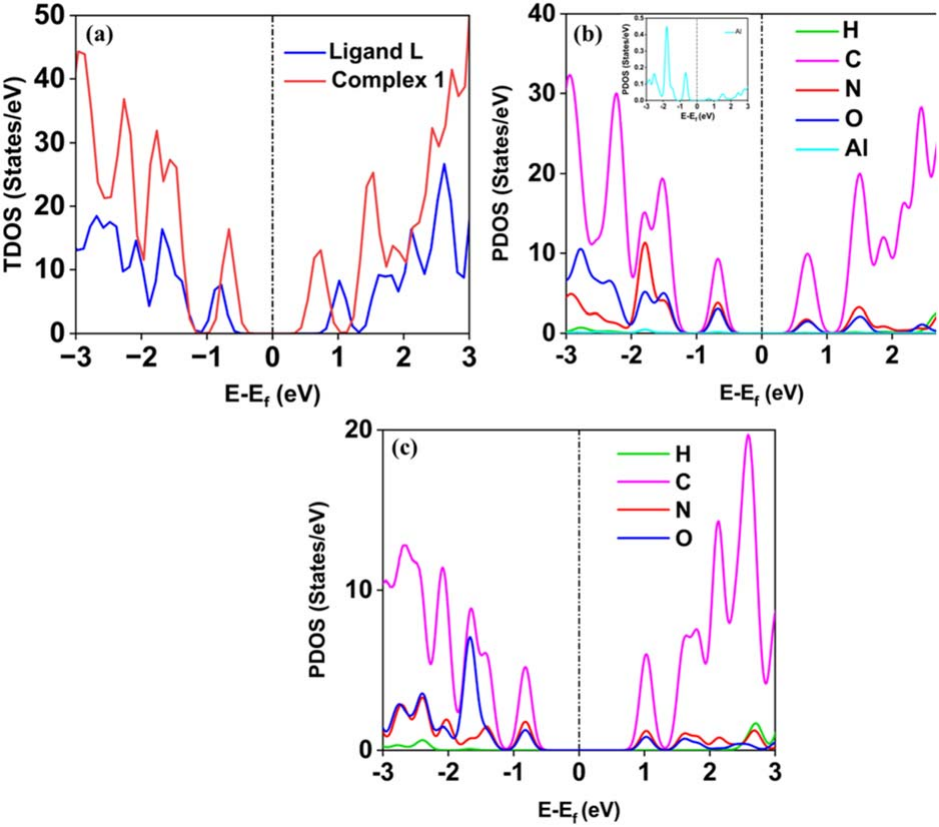
(a) Total density of states (TDOS) spectrum of ligand L and complex 1, the partial density of states (PDOS) spectrum of (b) complex 1, and (c) ligand L. The black dotted line indicates the Fermi level (*E*_F_).

The obtained experimental band gap demonstrates that the material belongs to the semiconductor family, which is also confirmed by the density of states (DOS) calculation as shown in Fig. 10. The DOS spectrum shows the presence of a significant gap between the valence band (VB) and conduction band (CB) region, indicating the semiconductor nature of ligand L and complex 1. Further, it was observed that complex 1 showed improved conductivity (band gap of less than 1 eV) as compared to ligand L (greater than 1 eV). Additionally, the computed PDOS indicates the contributions of individual atoms to the electronic behavior of the ligand L and complex 1. Interestingly, it was found that the individual atom contribution of complex 1 was higher than that of the ligand L.

## 7. Conclusions

In conclusion, we measured the electrical conductivity of a biphenyl derived amido Schiff base ligand L and its dinuclear Al(iii) complex (complex 1). It was found that the conductivity of complex 1 is two orders of magnitude higher compared with that of ligand L (conductivity of L, 1.05 × 10^−7^ Sm^−1^: and complex 1, 1.04 × 10^−5^ Sm^−1^). This increase in conductivity might be due to complex 1 extending itself to the polymeric supramolecular array by virtue of hydrogen bands, C–H⋯π(C) bonds and π⋯π interactions. The optical band gaps of the complex 1 and ligand L in the solid state were also determined experimentally from Tauc's plots and are found to be 2.63 eV and 3.04 eV, respectively. These values match well with the theoretical values of 2.53 eV and 3.07 eV, respectively, obtained from DFT calculations. Therefore, based on the band gap measured and theoretical calculations, we can predict that complex 1 could be used as a Schottky barrier diode (SBD).

## Data availability

Crystallographic data for ligand L and complex 1 have been deposited at the CCDC under 2260347 and 2260348, respectively, and can be obtained from https://doi.org/10.1039/d3dt01512b.

## Conflicts of interest

The authors declare no competing financial interest.
